# Phenotype of *Arabidopsis thaliana* semi-dwarfs with deep roots and high growth rates under water-limiting conditions is independent of the *GA5* loss-of-function alleles

**DOI:** 10.1093/aob/mcv099

**Published:** 2015-07-07

**Authors:** Luis Barboza-Barquero, Kerstin A. Nagel, Marcus Jansen, Jonas R. Klasen, Bernd Kastenholz, Silvia Braun, Birgit Bleise, Thorsten Brehm, Maarten Koornneef, Fabio Fiorani

**Affiliations:** ^1^Department of Plant Breeding and Genetics, Max Planck Institute for Plant Breeding Research, Cologne, Germany,; ^2^CIGRAS, Universidad de Costa Rica, San José, Costa Rica and; ^3^IBG-2: Plant Sciences, Forschungszentrum Jülich GmbH, Jülich, Germany

**Keywords:** *Arabidopsis thaliana*, semi-dwarfs, phenotyping, gibberellin biosynthesis, GA5, root system, low water availability, drought tolerance, GROWSCREEN

## Abstract

**Background and Aims** The occurrence of *Arabidopsis thaliana* semi-dwarf accessions carrying inactive alleles at the gibberellin (GA) biosynthesis *GA5* locus has raised the question whether there are pleiotropic effects on other traits at the root level, such as rooting depth. In addition, it is unknown whether semi-dwarfism in arabidopsis confers a growth advantage under water-limiting conditions compared with wild-type plants. The aim of this research was therefore to investigate whether semi-dwarfism has a pleiotropic effect in the root system and also whether semi-dwarfs might be more tolerant of water-limiting conditions.

**Methods** The root systems of different arabidopsis semi-dwarfs and GA biosynthesis mutants were phenotyped *in vitro* using the GROWSCREEN-ROOT image-based software. Semi-dwarfs were phenotyped together with tall, near-related accessions. In addition, root phenotypes were investigated in soil-filled rhizotrons. Rosette growth trajectories were analysed with the GROWSCREEN-FLUORO setup based on non-invasive imaging.

**Key Results** Mutations in the early steps of the GA biosynthesis pathway led to a reduction in shoot as well as root size. Depending on the genetic background, mutations at the *GA5* locus yielded phenotypes characterized by decreased root length in comparison with related wild-type ones. The semi-dwarf accession Pak-3 showed the deepest root system both *in vitro* and in soil cultivation experiments; this comparatively deep root system, however, was independent of the *ga5* loss-of-function allele, as shown by co-segregation analysis. When the accessions were grown under water-limiting conditions, semi-dwarf accessions with high growth rates were identified.

**Conclusions** The observed diversity in root system growth and architecture occurs independently of semi-dwarf phenotypes, and is probably linked to a genetic background effect. The results show that there are no clear advantages of semi-dwarfism at low water availability in arabidopsis.

## INTRODUCTION

Bioactive gibberellins (GA) are plant growth regulators responsible for the expression of several plant traits (Sun, 2008). Their biosynthesis and signalling pathways are well understood (Yamaguchi, 2008; Hedden and Thomas, 2012). Gibberellin-related mutations have played an important role in crop breeding because mutations in the GA signalling and biosynthesis pathways induce semi-dwarfism. These mutants have contributed to yield increases, particularly in wheat and rice, leading to the green revolution (Hedden, 2003; Salamini, 2003), especially by conferring lodging resistance. Semi-dwarf mutants that were selected by plant breeders had no negative pleiotropic effects (Sasaki *et al*., 2002; Spielmeyer *et al*., 2002; Jia *et al*., 2011) on yield-related traits and may have positively acting pleiotropic effects, such as those on the root system. However, effects on root growth are more difficult to measure systematically and quantitatively and this aspect has received little attention.

Root growth is regulated by plant hormones, and the role of auxins has been studied in detail (Overvoorde *et al*., 2010). Auxin gradients control the length of the primary root, the number of lateral root primordia and the response to gravity (Overvoorde *et al*., 2010). A recent review showed that GA could also regulate root elongation and thickening in the elongation zone of roots (Tanimoto, 2012). In that paper the author collected examples showing the sensitivity of shoots, hypocotyls and roots to GA and GA inhibitors and provides arguments supporting the view that roots have a higher GA sensitivity compared with shoots. In *Arabidopsis thaliana*, application of GA to the shoot increases the elongation of primary roots (Bidadi *et al*., 2010). Interestingly, this study records that application of GA inhibitors to the shoot also increases root elongation. This is probably due to negative feedback of GA levels on de *novo* synthesis (reviewed in Yamaguchi, 2008). Studies in *Populus* showed that GA-deficient mutants (with increased expression of *GA2ox1*) and GA-insensitive mutants (overexpression of *RGL1*) increase lateral root density and elongation (Elias *et al*., 2012). Crosstalk with the auxin hormone pathway may occur (Gou *et al*., 2010).

Several naturally occurring arabidopsis accessions carrying non-functional *ga5* alleles were identified (Barboza *et al*., 2013; Luo *et al*., 2015). The *GA5* gene encodes GA 20-oxidase1 (a GA biosynthesis enzyme), causing semi-dwarfism when mutated (Koornneef and van der Veen, 1980; Xu *et al*., 1995). The occurrence of semi-dwarfism in nature indicates that positive selection in certain populations may confer an advantage under specific environmental conditions. Recent studies suggest that semi-dwarfism is involved in adaptation to high altitudes (Luo *et al*., 2015). However, this may not be the only reason why semi-dwarfism occurs, because this phenotype is present in many coastal (low-altitude) regions of the Netherlands and Scandinavia (Barboza *et al*., 2013). *GA5* is the functional orthologue of the *SD1* rice gene and of the *Sdw*/*Denso* barley gene. Mutations in both genes lead to semi-dwarfism in modern rice and barley cultivars. Modifications of root growth can have an impact on the growth performance of plants under stress conditions and genetic variation in the hormonal pathways may affect root traits (Ghanem *et al*., 2011). There is experimental evidence that modern semi-dwarf barley genotypes have a longer root system compared with non-dwarf ones and quantitative trait locus (QTL) alleles controlling plant height co-localize with QTLs for root system size (Chloupek *et al*., 2006). Furthermore, dwarf alleles play a role in nitrogen acquisition under low nutrient supply, since it has been found that the *sdw1* dwarf allele in barley has a smaller root mass under reduced nutrient supply compared with long-stem varieties (Karley *et al*., 2011). A study by Vartanian *et al.* (1994) using arabidopsis showed that the *ga5* mutant has a drought-stress-adapted root system that might be more effective than the corresponding wild type in water acquisition. This was measured in water-withholding pot experiments studying the number of short roots present along lateral root axes (Vartanian *et al*., 1994). It remains to be established under which water-limiting scenario this trait can be beneficial. Moreover, recent studies showed the important role of the *DEEPER ROOTING 1* (*DRO1*) mutation in rice, which increased rooting depth (>2-fold in Dro1-NIL compared with its genetic background, IR64) and conferred drought tolerance (Uga *et al*., 2013). Deeper roots may be advantageous in water capture from the subsoil in dry environments. To our knowledge, there are no studies describing the root system of natural arabidopsis semi-dwarfs or growth performance under water-limiting conditions.

The aim of our study was to (1) characterize the shoot and root system of GA biosynthesis mutants and selected arabidopsis accessions carrying functional and mutated alleles of the *GA5* gene, and (2) to evaluate their response to reduced water availability. If mutations at the *GA5* locus have positive pleiotropic effects on root growth, this could help explain their selective advantage in specific environments, with particular reference to reduced water availability.

## MATERIALS AND METHODS

### Plant material

The selection of genotypes of *Arabidopsis thaliana* used in these experiments (Table 1) is based on previous studies (Barboza *et al*., 2013). For the QTL mapping experiment, the L*er* × Kas-2 recombinant inbred line (RIL) population was used (El-Lithy *et al*., 2005). In previous experiments, all plants were propagated by single-seed descent together in a greenhouse at the Max Planck Institute for Plant Breeding Research, Germany.

**Table 1.** List of genotypes included in the research

Genotype	Type	Height phenotype	Reference/collector
Kas-2	Accession	Semi-dwarf	Sharma
Kas-0	Accession	Wild type	Sharma
Kl-2	Accession	Semi-dwarf	Hülsbruch
Je-0	Accession	Wild type	Platt *et al*., 2010
Pak-3	Accession	Semi-dwarf	J. Mirza
Pak-1	Accession	Wild type	J. Mirza
OW-0	Accession	Semi-dwarf	M. Koornneef
OW-1	Accession	Wild type	M. Koornneef
Sparta	Accession	Semi-dwarf	M. Nordborg
T620	Accession	Wild type	Platt *et al*., 2010
Dja-1	Accession	Semi-dwarf	O. Loudet
Neo-3	Accession	Wild type	O. Loudet
Col	Accession	Wild type	N907^a^
L*er*	Accession	Wild type	NW20^a^
*ga20ox2-1*	Mutant (Col)	Wild type	Rieu *et al*., 2008
*ga20ox1-3*	Mutant (Col)	Semi-dwarf	Rieu *et al*., 2008
*ga20ox1 ga20ox2*	Double mutant (Col)	Semi-dwarf	Rieu *et al*., 2008
*ga1-13*	Mutant (Col)	Dwarf	Alonso *et al*., 2003
*ga1-3 6xbxcol*	Mutant (Col)	Dwarf	Mitchum *et al*., 2006
*ga3ox1-3*	Mutant (Col)	Semi-dwarf	Mitchum *et al*., 2006
*ga3ox1-3 ga3ox2-1*	Double mutant (Col)	Dwarf	Mitchum *et al*., 2006
*gai*	Mutant (L*er*)	Semi-dwarf	Koornneef *et al*., 1985
*ga4*	Mutant (L*er*)	Semi-dwarf	Koornneef and van der Veen, 1980
*ga5*	Mutant (L*er*)	Semi-dwarf	Koornneef and van der Veen, 1980

^a^Nottingham Arabidopsis Stock Center (NASC) identification number.

### *In vitro* root experiments

In a first approach, to understand the role of different mutations in the GA biosynthesis and signalling pathway, different mutants (Table 1) were phenotyped. All experiments were conducted in a complete randomized design. In a second approach, to characterize the phenotype of the semi-dwarf accessions an experiment was conducted with 12 natural accessions (Table 1) together with the *ga5* mutant and its parental wild type, L*er*. L*er* and the *ga5* mutant were included in all experiments. Experiments were performed from 11 June 2012 to 13 July 2012. Root systems were phenotyped *in vitro* using the GROWSCREEN-ROOT system with shoots growing outside the agar plate and roots growing within the agar medium (Nagel *et al*., 2009). The culture medium contained one-third Hoagland solution and 1 % agar as described in previous studies (Nagel *et al*., 2009). Plants were grown on 120 × 120 × 17-mm plates (Greiner) filled with ∼ 166 mL of medium. Seeds were sterilized with 70 % ethanol (3 min) followed by 0·5 % NaOCl (10 min), and were then rinsed three times with sterile Milli-Q H_2_O. After sowing, seeds were incubated at 4 °C in the dark for 5 d. Subsequently, the plates were incubated vertically in a growth cabinet (Bioline VB 1100 Vario; Vötsch Industrietechnik, Germany) set to an 8/16-h light/dark period, 22 °C day/18 °C night temperature and 60 % air humidity. Each plate contained four plants of the same genotype and there were three replications (*n*  =  12). Plants were phenotyped and shoots were harvested and immediately weighed to obtain the fresh weight at day 18 after germination (during the morning, 0900–1200 h). Dry weight of shoots was determined after samples had been oven-dried at 70 °C for ∼48 h or until constant weight was reached. The evaluated root and shoot traits are summarized in Table 2.

**Table 2.** Traits evaluated in the experiments conducted *in vitro*

Abbreviation	Trait	Quantification method
LLR	Length lateral roots	Nagel *et al*., 2009
LPR	Length primary root	Nagel *et al*., 2009
RSD	Root system depth	Nagel *et al*., 2012
RSW	Root system width	Nagel *et al*., 2012
TRL	Total root length	Nagel *et al*., 2009
PLA	Projected leaf area	Walter *et al*., 2007
SDW	Shoot dry weight	Gravimetric
SFW	Shoot fresh weight	Gravimetric
RSR	Root to shoot ratio	RSD/SFW
LDMC	Leaf dry matter content	SDW/SFW
SLA	Specific leaf area	PLA/SDW
PLT	Proxy leaf thickness	SLA × LDMC

Additional experiments were conducted to measure root system depth (RSD) in the L*er* × Kas-2 RIL population (El-Lithy *et al*., 2005) and in *F*_2_ lines derived from the cross L*er* × Pak-3. Plants were grown in half-strength Murashige and Skoog medium (Duchefa), pH 5·8, 0·8 % agar (Plant Agar; Duchefa), 100 µL L^−1^ Plant Preservative Mixture (PPM). In all experiments, seeds were stratified for 5 d at 4 °C in the dark. The plates used were the same as those described above. In this case plants were grown inside the plate, with six plants per plate. Experiments were conducted from 12 April 2013 to 3 May 2013. After stratification, seeds were transferred to a growth chamber (KL 01 U KTG MC; Van den Berg Klimaattechniek, The Netherlands) at 25/20 °C (day/night). Plants were phenotyped with a digital calliper (Series 500, 0–200 mm; Mitutoyo, Japan) at day 21 after germination (during the morning, 0900–1200 h).

The mutants *ga1-13*, *ga1-3* 6xbxcol and *ga3ox1-3 ga3ox2-1* were stratified in 100 µm GA_4+7_ solution (Duchefa) to ensure germination under the same temperature conditions as those described above. The GA stock solution was at a concentration of 25 mm (GA dissolved in a few drops of 1 m KOH).

### Rhizotron experiments

An experiment was conducted with six arabidopsis accessions, the *ga5* mutant and L*er*. Plants were grown in rhizotrons (60 × 30 × 2 cm) filled with peat soil (Graberde; Plantaflor Humus, Vechta, Germany; containing N, ∼120 mgL^–1^; P_2_O_5_, ∼20 mgL^–1^; K_2_O, ∼170 mgL^–1^), as described in previous studies (Nagel *et al*., 2012). Seeds were sown directly onto the surface of the soil-filled rhizotrons and stratified for 4 d at 4 °C (18 February 2013). Afterwards, plants were grown at 23 ± 10 °C, 53 ± 33 % relative humidity in the PhyTec greenhouse of the Institute of Plant Sciences (IBG-2) at Forschungszentrum Jülich, Jülich, Germany. One plant per rhizotron was grown. Each genotype was tested with six repetitions. To account for microclimate variation, a randomized spatial layout was used in these experiments. The plants were supplied with tap water (electrical conductivity ∼440 mS cm^–1^) twice per week. The amount of irrigation water was adjusted taking into account plant age (60 mL until a plant age of 2 months, 100 mL in the third month after sowing). Root system depth was quantified by image processing using ImageJ (Schneider *et al*., 2012). Images were acquired once a week, during the morning (0900–1200 h, starting 7 March 2013 and finishing 10 May 2013) using a single-lens reflex camera (Canon EOS 40D, Canon EF 24–70 mm zoom lens).

### Above-ground phenotyping experiments

Water-withholding experiments were conducted using pot-grown plants measured with the GROWSCREEN-FLUORO setup (Jansen *et al*., 2009) built into a climate-controlled growth chamber (SCREEN chamber) and integrated into an automated plant evaluation routine. For plant cultivation, single seeds were sown in 576-well trays filled with soil (Pikiererde; Balster Einheitserdewerk, Fröndenberg, Germany). Stratification was conducted for 4 d at 4 °C. The trays were then moved to the growth chamber (8/16-h light/dark period, 22 °C day/18 °C night temperature, air humidity 50 %). After cotyledon unfolding, seedlings were transplanted to pots (7 × 7 × 8 cm) filled with peat–sand–pumice substrate (SoMi 513 Dachstauden; Hawita, Vechta, Germany). Pots were randomized and arranged in trays (40 pots per tray). Pots of both control (well-watered) and treated (water-withholding) groups were water-saturated upon transplanting, reaching a volumetric soil water content (cm^3^/cm^3^) of ∼60 %. During seedling establishment after transplantation, the pots were progressively dried until reaching 30 % volumetric soil water content. They were maintained at this moisture level until the start of the water-withholding treatment based on gravimetric measurements and adjustment of the irrigation schedule (week 4). Pots of the control group were kept at this moisture level for the rest of the experiment, whereas the treated group was not irrigated until volumetric soil water content reached ≤6 %. At this soil moisture level the plants stopped growing, as determined by analysis of the projected rosette area (Jansen *et al*., 2009). Re-watering was then done (week 6) up to 30 % volumetric soil water content of the control group and plants were allowed to recover for 1 week. Based on the analysis of the water retention curves conducted in 2013 at the University of Kiel, Institute of Plant Nutrition and Soil Science, Germany, the corresponding estimated water potential of the soil used for this experiment was 0 MPa at 60 % cm^3^/cm^3^, –0·03 MPa at 30 % cm^3^/cm^3^ and less than –1·5 MPa, i.e. below the permanent wilting point, at 6 % cm^3^/cm^3^. These water potential values were estimated using eight-point curves that were fitted with the van Genuchten equation (van Genuchten 1980).

The first experiment was conducted with a group of GA biosynthesis and signalling mutants (conducted from 26 July 2012 to 14 September 2012). In the second experiment, natural accessions together with *ga5* and L*er* were studied (conducted from 7 September 2012 to 26 October 2012). Each genotype had 10–20 repetitions (depending on the experimental setup). A complete randomized design was used. Gravimetric monitoring of tray water content, water supply and GROWSCREEN FLUORO data acquisition and shoot trait evaluation were conducted as described in previous studies (Jansen *et al*., 2009). Projected leaf area measurements took place five times per week; upon each measurement the positions of the trays in the growth room were changed in order to compensate for position effects. On the last day of evaluation the shoots were harvested and immediately weighed to obtain the fresh weight (during the morning, 0900–1200 h). Dry weights of shoots were determined as described above. The mutants *ga1-13*, *ga1-3* 6xbxcol and *ga3ox1-3 ga3ox2-1* were germinated as described for the *in vitro* root experiments.

### Data analysis

Descriptive statistics and correlations were calculated and ANOVA was performed with R software (R Core Team, 2014). *Post hoc* Tukey tests were conducted using the R package Agricolae (de Mendibur, 2014). Principal components analysis (PCA) was done with the package FactoMineR (Lê *et al*., 2008). To estimate the area under the curve (AUC) for the projected leaf areas, the R package MESS (Ekstrom, 2014) was used. In order to estimate confidence intervals for the AUC ratios, 1000-fold bootstrapping of the AUC estimation was performed, using the R package boot (Canty and Riple, 2014). The R/qtl package was used to map QTL (Broman *et al*., 2003; Arends *et al*., 2010). The function mqmscan was used, setting all markers as cofactors, with further processing by backward elimination. To quantify the explained variance, QTLs above the 5 % significance threshold (using 1000 permutations) were selected and fitted into a multiple QTL model using the function fitqtl based on the Haley–Knott regression method.

## RESULTS

### Gibberellin biosynthesis mutants exhibit reduced shoot and root development *in vitro*

**Fig. 1.**
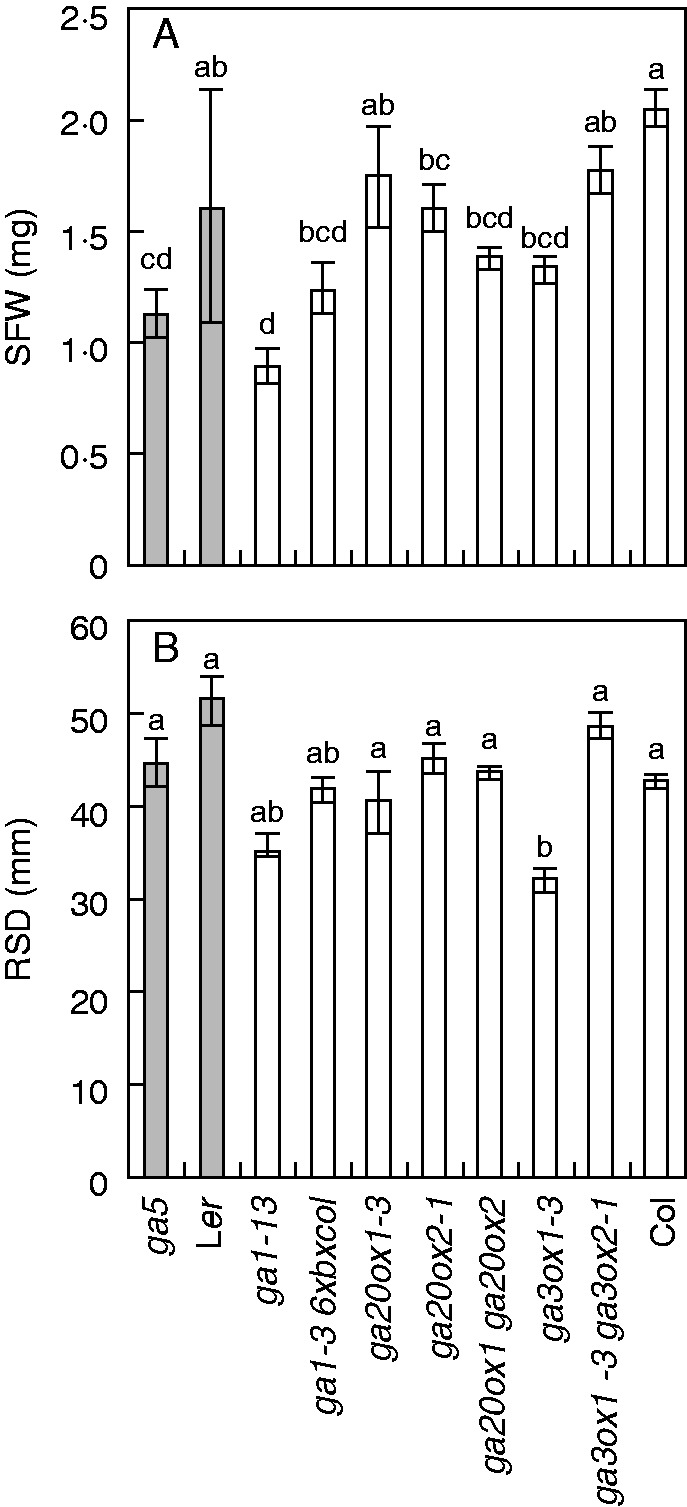
Phenotypes of arabidopsis GA biosynthesis mutants. Means ( ± s.e., *n* = 4–12) of (A) shoot fresh weight (SFW) and (B) root system depth (RSD) in different GA biosynthesis mutants and their corresponding wild-type controls, grown *in vitro* (1 % agar with 1/3 Hoagland solution, evaluated 18 d after germination). All mutants are on the Col background except *ga5*, which has the L*er* background. Different shadings indicate near-isogenic comparisons. The letters above each column indicate the results of a *post hoc* Tukey’s HSD test; means with different letters are significantly different (*P* < 0·05).

To evaluate the role of mutations in the GA biosynthesis pathway in the modulation of root growth, different GA-deficient mutants were phenotyped *in vitro* using the agar-based GROWSCREEN-ROOT assay system (Nagel *et al*., 2009). Semi-dwarf mutants in the *GA20ox1* (*GA5*) locus were included in the two backgrounds, L*er* (*ga5*) and Col (*ga20ox1-3*, *ga20ox1 ga20ox2*). Additional mutants with semi-dwarf (*ga3ox1-3*) and extreme dwarf (*ga1-13*, *ga1-3 6xbxcol*) phenotypes were included. All mutants exhibited decreased shoot fresh weight (SFW) at day 18, except *ga20ox1-3* and *ga3ox1-3 ga3ox2-1*, which had a phenotype comparable to that of their backgrounds (Fig. 1A). None of the mutants showed a significant decrease in RSD, or even any change in RSD, except *ga3ox1-3* (Fig. 1B). The *ga5* mutant mainly showed a significant decrease in SFW (Fig. 1A), projected leaf area (Supplementary Data Fig. S1) and proxy leaf thickness (calculated by multiplying specific leaf area by leaf dry matter content; Table 2, Supplementary Data Fig. S1). The results indicated that the *GA 20-oxidase 1* (*ga5*) mutation leads to a more pronounced growth reduction in terms of SFW on the L*er* background compared with the Col background because differences between *ga5* and L*er* were greater (29·7 % reduction compared with wild type) than those observed between *ga20ox1-3* and Col (15·0 % reduction compared with wild type). These findings were supported by PCA using all evaluated traits, in which *ga20ox1-3*, *ga20ox2-1* and *ga20ox1 ga20ox2* tended to cluster near their background accession Col while the *ga5* mutant was further from its wild-type genetic background, L*er* (Supplementary Data Fig. S2).

### Root phenotype of semi-dwarf accessions depends on the genetic background

**Fig. 2.**
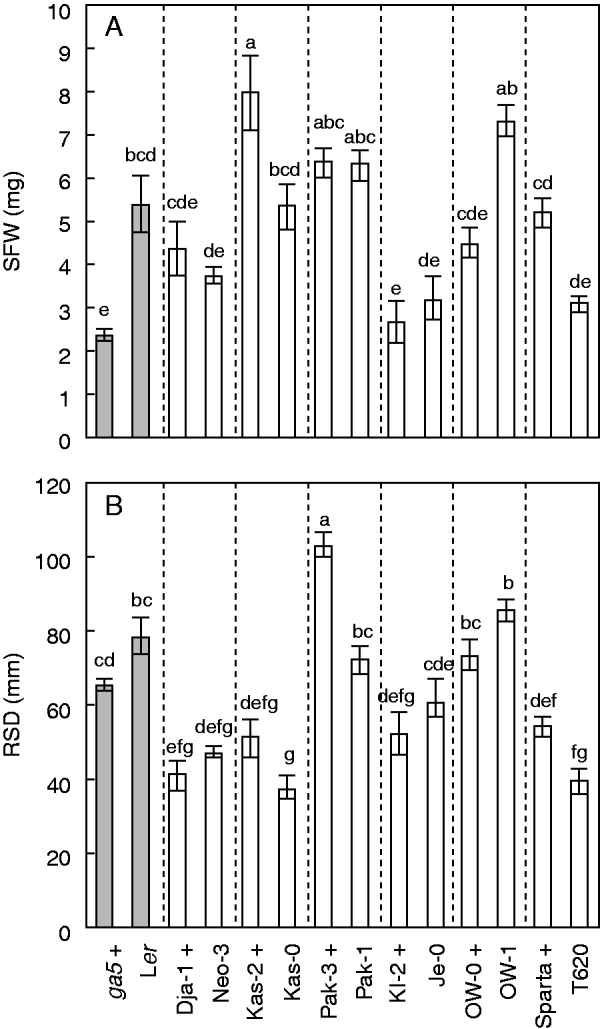
Contrasting phenotypes in the semi-dwarf accessions Pak-3 and Kas-2. (A) Shoot fresh weight (SFW) and (B) root system depth (RSD) (mean ± s.e., *n* = 6–12) in 13 arabidopsis accessions and the *ga5* mutant (L*er* background) grown *in vitro* (1 % agar with 1/3 Hoagland solution, evaluated 18 d after germination). Semi-dwarf phenotypes are indicated by ‘+’ after the accession name. The letters above each panel indicate the results of *post hoc* Tukey’s HSD test; means with different letters are significantly different (*P* < 0·05). Vertical dotted lines separate near-related accessions comparisons.

We characterized the root system architecture of several semi-dwarf accessions allelic to *ga5* (Barboza *et al*., 2013) and related non-dwarf genotypes. Variation among accessions carrying active and inactive *ga5* alleles was observed, suggesting no consistent effect of semi-dwarfism for the evaluated traits. The semi-dwarf accession Kas-2 had the highest SFW value, while the semi-dwarf accession Kl-2 had the lowest (Fig. 2A). The semi-dwarf Pak-3 showed the longest RSD and the non-dwarf Kas-0 the shortest RSD (Fig. 2B). The PCA analysis showed no clustering of semi-dwarf accessions, suggesting that semi-dwarfism does not contribute *per se* to variation in the evaluated traits (Supplementary Data Fig. S3A). However, this PCA analysis indicated that Pak-3 is an outlier, mainly because of its deeper root system and the length of its primary root (Supplementary Data Fig. S3B). It was possible to test the semi-dwarf effect among the traits because we included a balanced number of accessions carrying active and inactive *ga5* alleles. Remarkably, no semi-dwarf effect was significant. Only root system width, representing the maximum horizontal distribution of a root system, was significantly different between dwarf and wild-type genotypes (*P* < 0·001; mean value for semi-dwarfs 9·0 ± 0·4 mm versus 12·8 ± 0·8 mm for wild type). Neo-3 showed an increased root system width compared with the other accessions (mean ± s.e. under control conditions 23·8 ± 1·6 mm versus 7·3 ± 1·0 mm for the semi-dwarf Dja-1, a genetically related accession). However, when the non-dwarf Neo-3 was excluded from the analysis the overall effect was not statistically significant (*P* > 0·05). These observations lead to the conclusion that shoot and root modifications depend on the genetic background, which might interact to some extent with the *ga5* genotype. Apparently, variation at additional loci contributes to genotype differences for the evaluated traits.

We used QTL analysis as an independent approach to test the possible role of *ga5* in controlling shoot biomass and RSD *in vitro* using the L*er* × Kas-2 population (El-Lithy *et al*., 2005). The advantage of using this population is that semi-dwarfism due to the Kas-2 loss-of-function allele at the *GA5* locus is segregating, which allows us to test the effect of *ga5* on RSD. Transgressive segregation was observed for SFW and RSD (Supplementary Data Fig. S4). When tested separately for each trait, *ga5* reduced both RSD and SFW, but this effect was relatively small and the log of the odds (LOD) score was not significant (Supplementary Data Figs S5 and S6). A locus at or near *ERECTA* (chromosome 2) and a locus located on chromosome 5 (SNP304) controlled mainly SFW (LOD scores 5·0 and 4·1) and the amount of variance explained was 14·3 and 10·6 %, respectively (Supplementary Data Fig. S6). The *ERECTA* locus might play a minor role in RSD, together with a QTL on chromosome 4 (M4-3), but no QTL position was detected for RSD in the vicinity of the *ga5* locus.

Pak-3, a semi-dwarf accession, showed the greatest rooting depth among all those tested (Fig. 2B). All observed phenotypes in the evaluated natural semi-dwarf accessions led to the conclusion that semi-dwarfism does not affect this trait. Nevertheless, indications that barley semi-dwarf accessions show a higher root length (Chloupek *et al*., 2006) raised the question of whether or not the long Pak-3 RSD is dependent on the *ga5* mutation. To test this hypothesis, a co-segregation analysis was conducted by phenotyping RSD and plant height in *F*_2_ populations derived from the hybrids *ga5* × Pak-3 and L*er* × Pak-3. The goal of this experiment was to quantify whether RSD and shoot height variation (due to *ga5* genotype) were correlated. Clear differences for both plant height phenotype and RSD were observed. However, these traits segregated independently of each other (Supplementary Data Fig. S7). We conclude that *ga5* does not play a role modifying RSD in the Pak-3 accession.

**Fig. 3.**
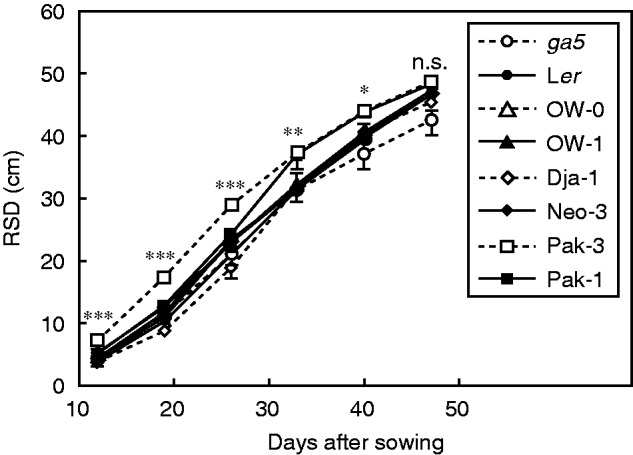
Root system depth (RSD) of soil-grown accessions. Development of RSD across time in seven arabidopsis accessions and the *ga5* mutant (L*er* background) grown in soil-filled rhizotrons. Data are mean ± s.e. (*n* = 3–6). Asterisks show *P* values from ANOVA to test for significant differences among genotypes: ***≤ 0·001, **≤ 0·01, *≤ 0·05, n.s. not significant (*P* > 0·05). Dashed lines represent genotypes with semi-dwarf phenotype. Similar marker styles indicate near-related accessions.

**Fig. 4.**
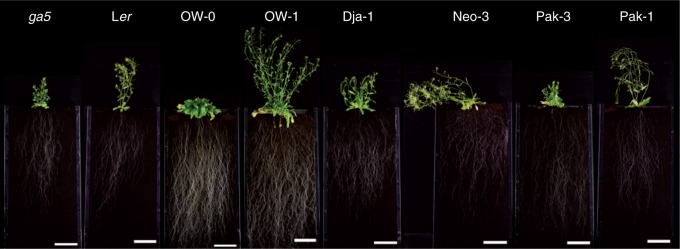
Arabidopsis accessions and the *ga5* mutant (L*er* background) grown in soil-filled rhizotrons. Photographs of representative individuals were taken 2 weeks after flowering. Near-related accessions are next to each other. Scale bar = 10 cm.

To further characterize the evaluated semi-dwarf accessions, a selected group of genotypes (*ga5*, L*er*, OW-0, OW-1, Dja-1, Neo-3, Pak-3, Pak-1) was phenotyped in rhizotrons using soil as substrate. As observed in the agar plate experiments, the Pak-3 accession had the greatest RSD 4 weeks after sowing (Fig. 3). In contrast, another Central Asian semi-dwarf accession, Dja-1, presented the smallest rooting depth, which did not differ significantly from that of the related wild type, Neo-3. The semi-dwarf mutant *ga5* showed a smaller RSD compared with its wild type, L*er*. A final evaluation was performed 2 weeks after flowering to phenotype these genotypes for plant height. As previously reported (Barboza *et al*., 2013), the *ga5* mutation only had a significant effect on average plant height (*P* < 0·0001), while differences in the root system at this stage were apparently independent of this locus (Fig. 4).

### Responses to reduced water availability

The presence of semi-dwarfs in nature indicates that this trait might confer a selective advantage under specific conditions, in particular in environments characterized by the occurrence of abiotic stresses. Previous studies suggested that *ga5* might be more drought-tolerant due to modifications of root system architecture (Vartanian *et al*., 1994). We quantified the performance of GA biosynthesis mutants and natural semi-dwarf accessions under reduced water availability. To create water stress in agar plates, an experiment using osmotic stress was conducted by applying sorbitol (100 mm). The percentage of growth (relative to control) for all evaluated traits was compared. No strong differences were observed when comparing growth percentage for the different evaluated GA mutants (Supplementary Data Table S1). Only leaf dry matter content (LDMC) was different when comparing *ga5* with L*er* (164·5 ± 21·0 versus 61·2 ± 30·4 %) and mutant *ga20ox1-3* with Col-0 (151·4 ± 22·7 versus 114·8 ± 6·4 %). Also, the double mutant *ga20ox1 ga20ox2* showed a higher LDMC growth percentage compared with its background accession Col-0 (150·1 ± 57·8 versus 114·8 ± 6·4 %). Different accessions were tested under the same conditions. This experiment did not show significant effects of semi-dwarfs compared with wild-type accessions (Supplementary Data Fig. S8). The semi-dwarf Dja-1 showed the best performance (112 % growth relative to control), indicating this accession was not affected by the sorbitol treatment.

**Fig. 5.**
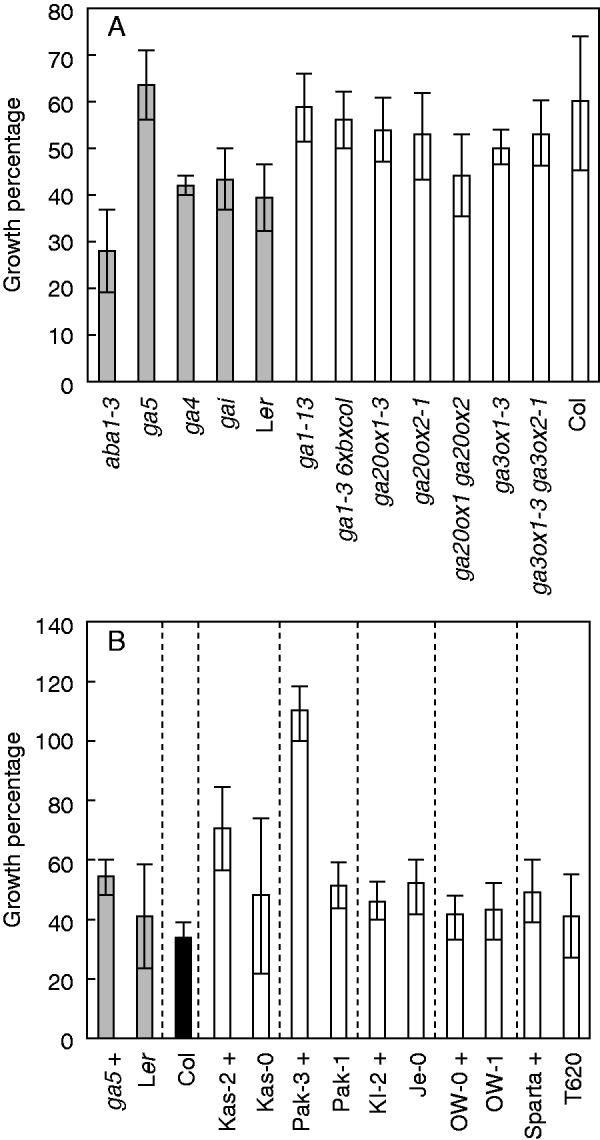
Water withholding and response (growth percentage under water-limiting conditions relative to control) among arabidopsis GA biosynthesis and signalling mutants and natural accessions. (A) Growth percentages for shoot fresh weight (SFW) representing variation among mutants and (B) natural accessions grown in soil on pots after withholding of water followed by 1 week of recovery after re-watering. Shoots were harvested at week 7. In (A), different shadings indicate near-isogenic comparisons. Growth percentages were obtained using mean values (water withholding/control) ± uncertainties (*n* = 4–14 for water withholding, *n* = 4–12 for control). Vertical dotted lines in (B) separate near-related accessions. Semi-dwarf phenotypes are indicated by ‘+’ after the accession name.

**Fig. 6.**
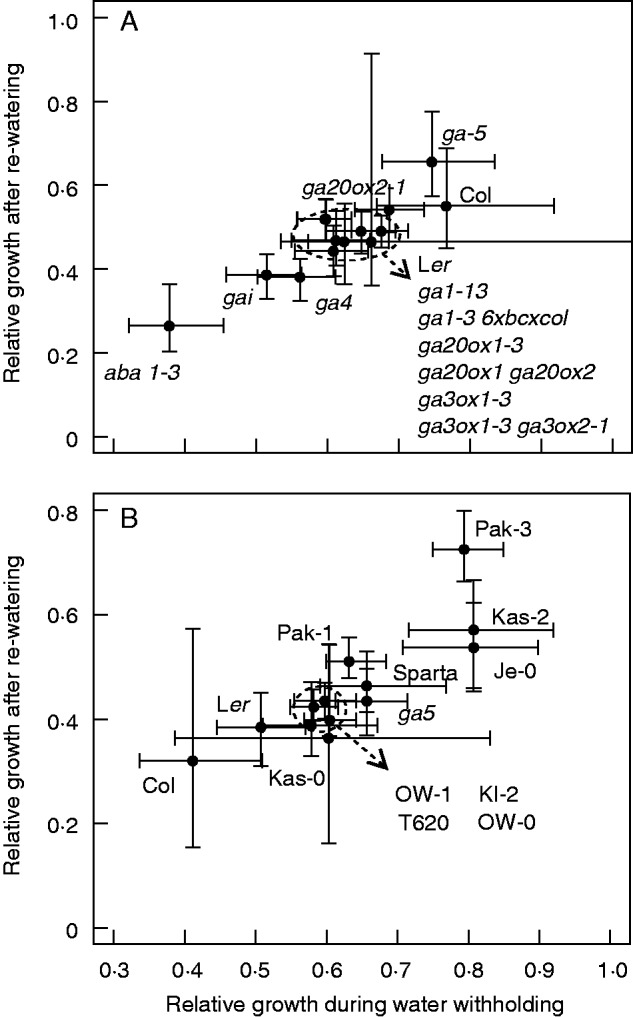
Water-withholding responses among arabidopsis (A) GA biosynthesis and signalling mutants, and (B) natural accessions grown in soil. Ratios were obtained by dividing the water-withholding value by the control value for the area under the curve for projected leaf area in the drought and recovery phases. Vertical and horizontal lines represent 95 % confidence intervals (*n* = 4–14 for water withholding, *n* = 4–12 for control).

**Fig. 7.**
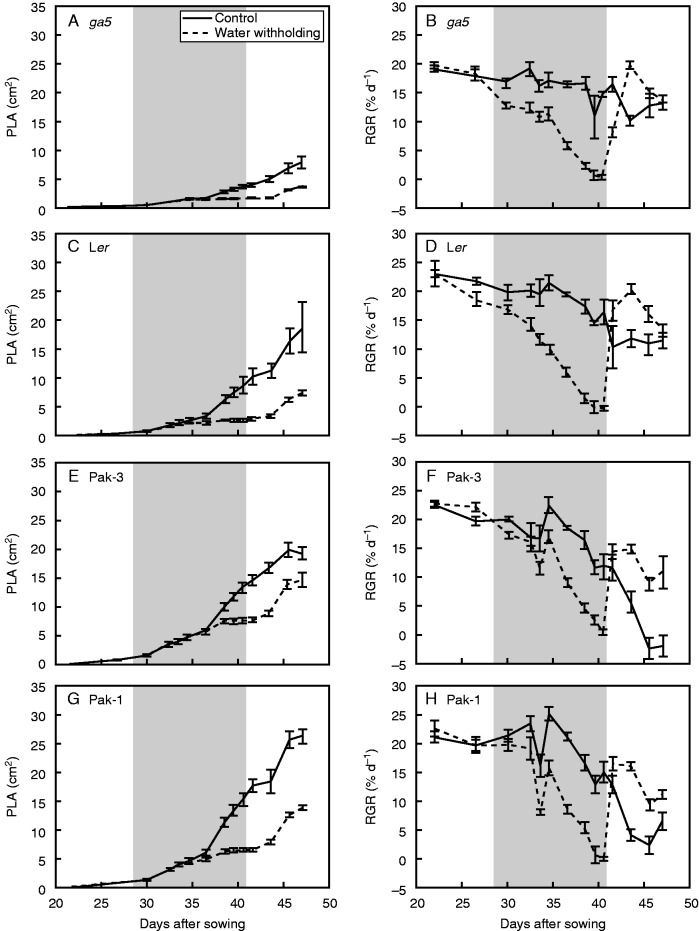
Growth responses of five arabidopsis accessions and the *ga5* mutant to water withholding. The traits illustrated are projected leaf area (PLA) (A, C, E, G) and relative growth rate (RGR) (B, D, F, H). RGR was estimated using PLA values. Plants were grown in soil on pots. Grey shading indicates the period of water withholding. Data are mean ± s.e. (*n* = 4–14 for water withholding, *n* = 4–12 for control).

To test a different water limitation scenario, water-withholding experiments using pots filled with soil were conducted. For this experiment the mutants *ga4* (encoding the GA biosynthesis enzyme GA3ox1, causing semi-dwarfism when mutated), *gai* (GA signalling mutant causing dwarfism when mutated) and *aba1-3* [abscisic acid (ABA)-deficient mutant; (Koornneef *et al*., 1982)] were included (all on the L*er* background). The mutant *aba1-3* showed the lowest performance for SFW under the water-limitation treatment (Fig. 5A). Among the induced mutants, *ga5* showed a higher growth percentage compared with its background accession L*er* for the trait SFW (Fig. 5A). However, the allelic *ga20ox1-3* mutant did not show differences from its background accession Col-0 (Fig. 5A). The double mutant *ga20ox1 ga20ox2* showed the lowest performance among the Col background accessions. Among the accessions, Pak-3 showed the highest growth percentage for SFW (Fig. 5B). The *ga5* mutant showed a better performance in SFW (in soil, water withholding) than L*er* (Fig. 5B). The use of GROWSCREEN-FLUORO (Jansen *et al*., 2009) allows the estimation of growth in time during and after drought using projected leaf area (PLA; Fig. 6). The output using PLA across time resembled the results obtained using SFW (Fig. 5A), in which the mutant *aba1-3* showed the lowest growth rate during both the water-withholding and the re-watering phase (Fig. 6A). Wide variation among the remaining genotypes (especially for L*er*) suggests a similar response, but it should be noted that the semi-dwarfs *gai* and *ga4* showed a moderate response (Fig. 6A). When this effect was evaluated in different accessions, the Pak-3 accession presented a high growth rate during water-limiting conditions, together with the semi-dwarf Kas-2 and the non-dwarf Je-0 (Fig. 6B). Besides, Pak-3 had the highest growth rate during the recovery phase (Fig. 6B). The *F*_1_ populations of the crosses *ga5* × Pak-3 and *ga5* × OW-0 showed no differences under water-limiting conditions compared with crosses of mutants with wild-type L*er* (data not shown). In this experiment (independently conducted), *ga5* tended to have a higher growth rate than L*er* (higher in the first experiment). Of note, *ga5* had a smaller PLA over time compared with L*er* (Fig. 7A, C), but despite this the relative growth rates (RGRs) of the two genotypes were similar (Fig. 7B, D). The differences between control and water-withholding treatments for the trait PLA tended be lower in *ga5* than in L*er* (Fig. 7A, C). The same was observed when comparing the semi-dwarf Pak-3 with the wild-type Pak-1 (Fig. 7E–H).

Taken together, these data show that this set of *ga5* alleles does not yield phenotypes that differ substantially in their response to reduced water availability compared with the corresponding wild-types, and therefore this mutation has no detrimental effect during water limitation. Both semi-dwarf and wild type genotypes maintain growth under reduced water supply, thus pointing to the involvement of additional loci in this response.

## DISCUSSION

*GA5* is a GA biosynthesis gene with a major effect only on plant height and without other major pleiotropic effects, as observed in mutants in the early steps of the GA pathway (Koornneef and van der Veen, 1980). Among all five AtGA20ox paralogues, *GA5* is the only one having a major effect on plant height when mutated, due to its expression pattern in comparison with that of the paralogues (Rieu *et al*., 2008; Plackett *et al*., 2012). Because the *ga5* mutants show no obvious trade-offs, we can explain why natural variants carrying loss-of-function alleles can be maintained in nature in contrast to mutations in early GA biosynthesis genes such as *ga1* (Barboza *et al*., 2013). Redundancy in the GA20ox genes might maintain GA homeostasis in all organs except the growing inflorescence stem and leaves, thus allowing *ga5* mutants to maintain a root system similar to that of the corresponding wild types. Despite this, the *ga5* mutant shows a moderate reduction of root length compared with the wild type, an effect observed in previous studies on the Landsberg *erecta* background (Vartanian *et al*., 1994), but not in Col (Rieu *et al*., 2008). This points to the genetic background playing a role, thus suggesting epistasis. A good example has been shown for the *SVP* gene, for which the same mutation has a different effect on flowering time depending on the genetic background (Méndez-Vigo *et al*., 2013).

Natural variation in root system architecture has been reported in arabidopsis. Several studies have used QTL analysis to map loci involved in root-related traits (Mouchel *et al*., 2004; Loudet *et al*., 2005; Fitz Gerald *et al*., 2005; Sergeeva *et al*., 2006). Other studies yielded mapping under different soil mineral concentrations showing the plasticity of the root system and the loci controlling these effects (Prinzenberg *et al*., 2010; Kellermeier *et al*., 2013). Genome-wide association studies (GWAS) showed that the genes *PHOSPHATE 1* (*PHO1*) and *Root System Architecture 1* (*RSA1*) were associated with root system allometry (Rosas *et al*., 2013). However, GWAS for total root length did not result in significant associations, suggesting the involvement of many loci in the control of this trait (Rosas *et al*., 2013). Another study of natural variation for root length identified loss-of-function alleles in the positive regulator of auxin signalling *BRX*, which modifies root length under acidic conditions (Gujas *et al*., 2012). These examples illustrate the role of specific root system architecture traits that may be selected for depending on the environment, as illustrated in a recent review (Lynch, 2013). This plasticity can affect different genes from independent pathways. The possible locus (loci) controlling RSD in the Pak-3 accession has not been mapped and therefore no conclusion can be drawn about whether the variation is due to known pathways or genes. The different root systems found in other Central Asian accessions, such as Kas-2 (relatively short root system) and Neo-3 (relatively large root system width), suggest that the variation present in this region and possible variable selective pressure may allow the evolution of specialized root systems. Our QTL analysis using the L*er* × Kas-2 RIL mapping population did not show that *ga5* controlled SFW or RSD. However, our QTL profile showed co-localization with QTLs mapped in previous studies using the same population for similar traits (Prinzenberg *et al*., 2010). The *ERECTA* locus in chromosome 2 may be a candidate controlling the main QTL for SFW, in agreement with previous studies (Prinzenberg *et al*., 2010). *ERECTA* encodes a receptor-like kinase (Torii *et al*., 1996) and loss-of-function alleles display reduced plant height, a compact inflorescence and rounder and smaller leaves, among other traits (Torii *et al*., 1996; van Zanten *et al*., 2009). Regarding the main QTL controlling RSD located on chromosome 4, Prinzenberg *et al.* (2010) mentioned that a sodium transporter gene located in this region may be implicated, although to our knowledge this has not yet been tested.

The successful use of semi-dwarfs in crops has been a topic for discussion regarding possible trade-offs under drought conditions. The rice variety IR64, carrying mutations in *GA20ox2* (Sasaki *et al*., 2002; Spielmeyer *et al*., 2002), the functional orthologue of *ga5* (*GA20ox1*), has been reported as drought-sensitive because it was bred for irrigated agricultural environments (Lafitte *et al*., 2007; Swamy *et al*., 2013). However, previous studies highlighted the occurrence of rice semi-dwarfs with drought tolerance (Lafitte *et al*., 2007). The physiological hypothesis behind this might be related to GA–ABA antagonism, whereby semi-dwarfs carrying low GA levels show ABA accumulation, which is beneficial under drought (Lafitte *et al*., 2007). As found in this study for arabidopsis, using PLA measurements in pot experiments under water-withholding conditions, semi-dwarf accessions may be tolerant (e.g. Pak-3). Even the isogenic comparison between *GA20ox* mutants, on both the Landsberg *erecta* and the Col background, show no trade-offs during drought.

Recent studies point to the relevance of RSD in increasing drought avoidance. Natural variants of rice with a deep rooting system have been isolated and the QTL associated with this trait has been identified as *DEEPER ROOTING 1* (*DRO1*) (Uga *et al*., 2013). Introgression of this gene into cultivated varieties conferred drought resistance. However, it is still disputed whether or not a long root system is directly translated into drought tolerance, as is argued for rice (Lafitte *et al*., 2007). A deep root system can be useful in soils containing deep water reservoirs. In this study we addressed the possible link between water-limiting conditions and rooting depth, such a link may be found in Pak-3, a semi-dwarf accession with a deep root system that showed high performance under water-limiting conditions. There is still the need to further understand and characterize the possible link between a long root system and its plausible selective advantage in nature and whether it displays possible trade-offs.

In conclusion, semi-dwarfism in arabidopsis does not show pleiotropic effects at the level of the root system that can be attributed to loss-of-function *GA5* alleles. We also conclude that there is no apparent positive advantage of semi-dwarfism under water-limiting conditions.

## SUPPLEMENTARY DATA

Supplementary data are available online at www.aob.oxfordjournals.org and consist of the following. Table S1: growth percentage under sorbitol treatment (relative to control) among arabidopsis GA biosynthesis mutants for different shoot- and root-related traits. Fig. S1: phenotypes of arabidopsis GA biosynthesis mutants. Fig. S2: principal components analysis and correlations for shoot- and root-related traits. Fig. S3: principal components analysis and correlations for shoot- and root-related traits. Fig. S4: frequency distribution for shoot fresh weight and root system depth in the L*er* × Kas-2 mapping population grown *in vitro*. Fig. S5: mean values of shoot fresh weight and root system depth in the L*er* × Kas-2 mapping population sorted by the parental alleles using the markers ERECTA, GA5 and SNP304. Fig. S6: QTL maps for shoot fresh weight and root system depth in the L*er* × Kas-2 mapping population grown *in vitro*. Fig. S7: long Pak-3 root system depth occurs independently of the *ga5* inactive allele. Fig. S8: osmotic stress response among arabidopsis accessions.

Supplementary Data
