# Supplementary material for: Phenotype of Arabidopsis thaliana semi-dwarfs with deep roots and high growth rates under water-limiting conditions is independent of the GA5 loss-of-function alleles
Source: Ann Bot. 2015 Jul 7;116(3):321–31. doi: 10.1093/aob/mcv099 (PMC4549960; doi:10.1093/aob/mcv099)
Supplement: Supplementary Data [file supp_116_3_321__index.html]

Phenotype of Arabidopsis thaliana semi-dwarfs with deep roots and high growth rates under water-limiting conditions is independent of the GA5 loss-of-function alleles — Phenotype of Arabidopsis thaliana semi-dwarfs with deep roots and high growth rates under water-limiting conditions is independent of the GA5 loss-of-function alleles — Supplementary Data 

# Phenotype of *Arabidopsis thaliana* semi-dwarfs with deep roots and high growth rates under water-limiting conditions is independent of the *GA5* loss-of-function alleles

## Supplementary Data

files

- Supplementary Data - pdf file
