# Supplementary material for: Phenotype of Arabidopsis thaliana semi-dwarfs with deep roots and high growth rates under water-limiting conditions is independent of the GA5 loss-of-function alleles
Source: Ann Bot. 2015 Jul 7;116(3):321–31. doi: 10.1093/aob/mcv099 (PMC4549960; doi:10.1093/aob/mcv099)

SUPPLEMENTARY DATA

Table S1. Growth percentage under sorbitol (relative to control) among *A. thaliana* GA biosynthesis mutants in different shoot and root related traits. Seedlings were grown *in vitro* (1% agar with 1/3 Hoagland solution, evaluated 18 days after germination). Values in parenthesis indicate the propagation of uncertainty (n = 5 – 12 for sorbitol, n = 6 – 12 for control). NA = Not applicable.

Genotypes	LPR	LLR	TRL	RSD	RSW	PLA	RSR	SFW	SDW	SLA	LDMC	PLT
ga5	71.8	99.1	76.7	71.8	112.8	81.8	101.9	69.9	114.0	70.7	164.5	118.7
	(5.3)	(34.4)	(9.5)	(5.4)	(16.1)	(10.0)	(12.7)	(9.4)	(9.9)	(6.5)	(21.0)	(13.3)
L*er*	72.7	43.2	65.4	72.8	62.4	88.7	61.8	91.1	73.6	112.5	61.2	93.2
	(7.6)	(24.1)	(10.6)	(7.6)	(22.9)	(26.3)	(26.7)	(32.4)	(13.5)	(26.1)	(30.4)	(11.9)
ga1-13	92.5	NA	85.9	92.5	91.7	81.4	118.1	76.8	86.4	82.5	114.8	105.6
	(5.3)	NA	(6.3)	(5.6)	(21.1)	(12.2)	(10.1)	(8.7)	(17.8)	(14.1)	(16.2)	(13.7)
ga20ox1-3	87.1	5.7	69.0	87.5	48.8	68.6	131.8	62.6	88.2	75.9	151.4	112.8
	(10.3)	(36.0)	(14.1)	(10.1)	(19.3)	(17.3)	(11.8)	(13.3)	(14.1)	(8.8)	(22.7)	(11.5)
ga20ox2-1	71.8	NA	63.8	71.0	45.0	59.3	107.0	65.9	73.6	80.9	107.6	83.4
	(5.0)	NA	(7.0)	(4.9)	(16.9)	(7.6)	(10.5)	(6.6)	(7.0)	(12.3)	(14.5)	(13.9)
ga20ox1 ga20ox2	77.9	NA	74.4	78.0	59.0	49.3	187.2	53.7	64.6	75.0	150.1	115.2
	(4.1)	NA	(4.4)	(4.0)	(16.8)	(10.6)	(99.5)	(3.3)	(6.7)	(11.3)	(57.8)	(41.4)
ga3ox1-3	77.5	20.1	70.7	78.1	48.6	67.3	98.3	80.7	96.1	70.6	120.3	83.2
	(6.4)	(25.1)	(7.8)	(6.6)	(13.9)	(10.7)	(6.6)	(5.7)	(8.0)	(9.4)	(13.2)	(8.8)
ga3ox1-3 ga3ox2-1	71.9	NA	68.0	71.9	68.0	44.1	109.0	66.7	74.3	60.9	110.2	67.1
	(4.3)	NA	(5.1)	(4.2)	(18.5)	(8.7)	(9.7)	(5.8)	(5.5)	(8.6)	(8.6)	(6.3)
Col	80.5	8.8	64.6	80.6	39.7	73.5	116.5	70.5	80.1	92.9	114.8	105.4
	(3.5)	(14.0)	(4.6)	(3.5)	(11.4)	(6.8)	(5.9)	(3.9)	(4.1)	(7.5)	(6.4)	(6.5)

Fig. S1. Phenotypes of *A. thaliana* GA biosynthesis mutants. Means (± standard errors, *n* = 4 - 12) of shoot and root traits in different GA biosynthesis mutants and their corresponding wild type controls, grown *in vitro* (1% agar with 1/3 Hoagland solution, evaluated 18 days after germination). All mutants are in Col background except *ga5,* which has the L*er* background. Different colours indicate near isogenic comparisons. The letters above each panel indicate the results of a *post-hoc* Tukey`s HSD test where means with different letters are significantly different ($P < 0.05$).

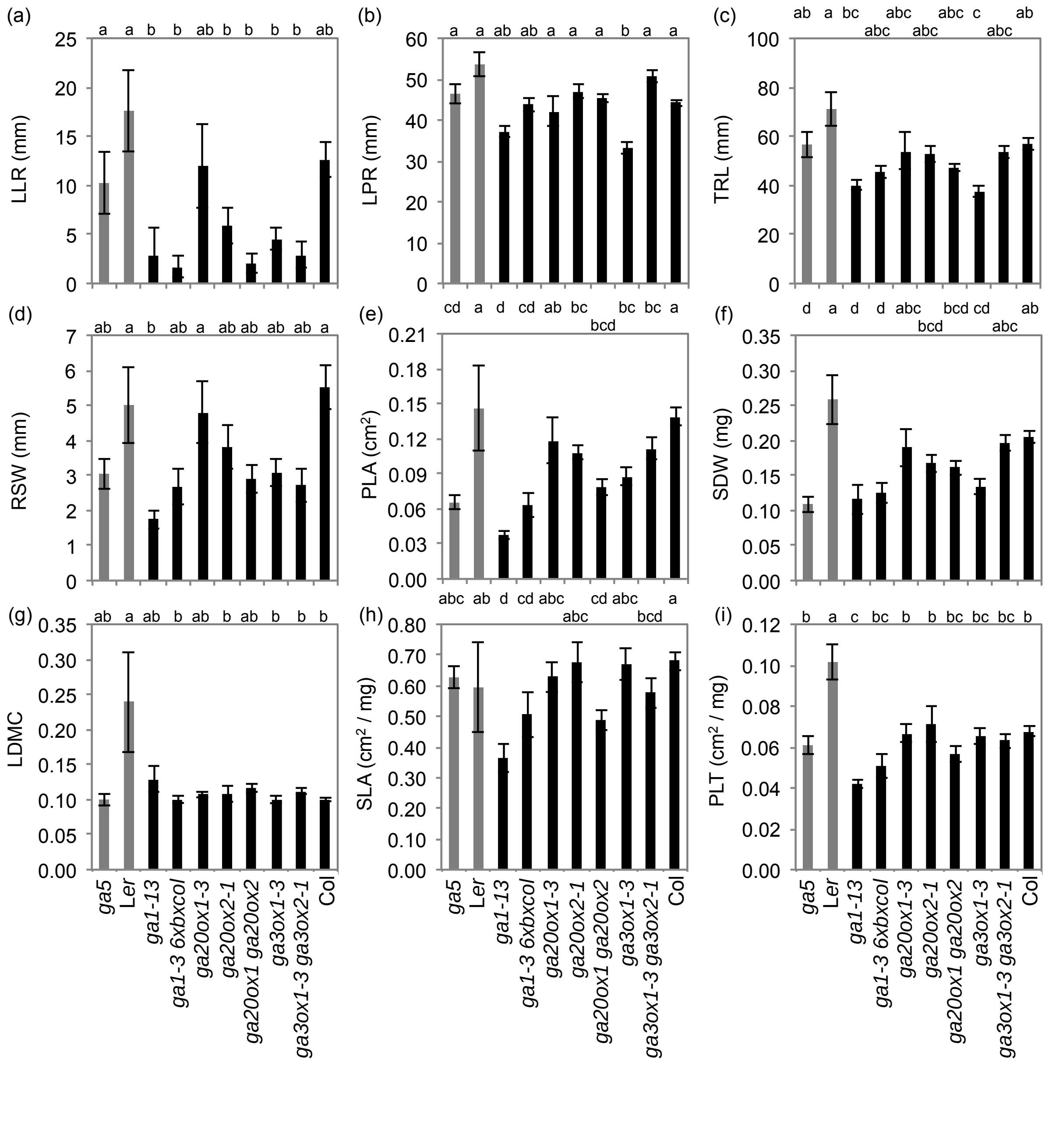

Fig. S2. Principal Component Analysis (PCA) and correlations for shoot and root related traits. PCA of GA biosynthesis *A. thaliana* mutants and their corresponding wild type controls, based on the combined data of different shoot and root phenotypes of plants grown *in vitro* (a; 1% agar with 1/3 Hoagland solution). Variables factor map (b) is shown. All mutants are in Col background except for *ga5*, which has L*er* background.

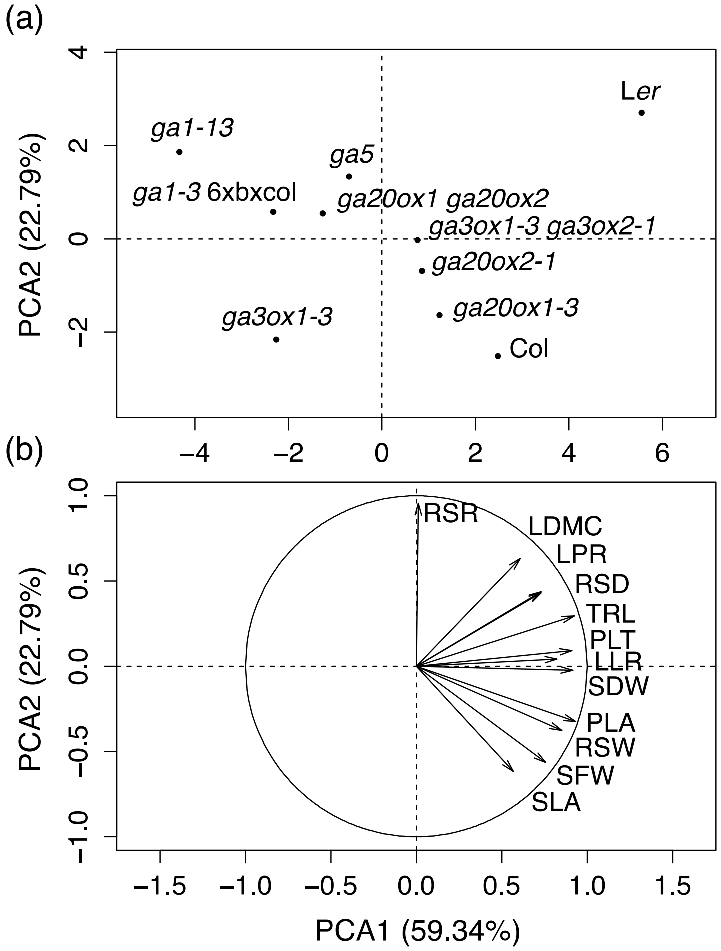

Fig. S3. Principal Component Analysis (PCA) and correlations for shoot and root related traits. PCA of 13 *A. thaliana* accessions and the *ga5* mutant (L*er* background), based on the combined data of different shoot and root phenotypes of plants grown *in vitro* (a; 1% agar with 1/3 Hoagland solution, evaluated 18 days after germination). Variables factor map (b) are shown. Accessions connected by dotted lines indicate genetically related pairs, e.g. *ga5* vs L*er* (*GA5*). The symbol ⊙ indicates semi-dwarf accessions.

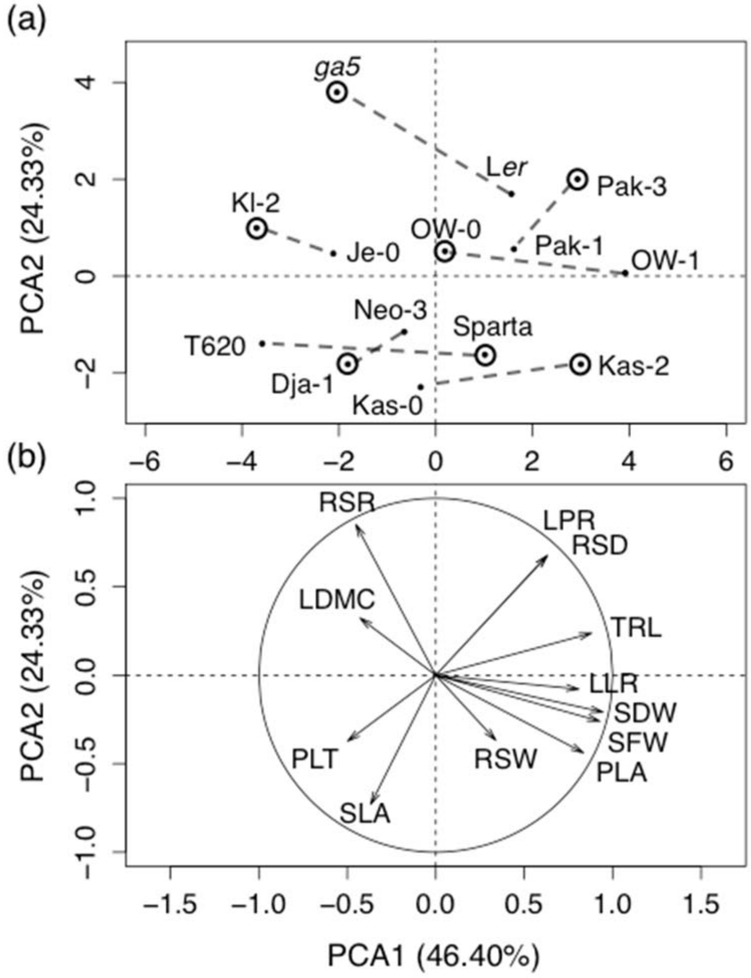

Fig. S4. Frequency distribution for shoot fresh weight (SFW) and root system depth (RSD) in the L*er* × Kas-2 mapping population grown *in vitro*. The SFW (a) and RSD (b) were evaluated in 125 recombinant inbred lines (RILs); six seedlings were sown per genotype. The medium contained: 0.8% Agar, 1/2 MS, pH 5.8. The experiment was evaluated 21 days after germination. The horizontal black bars denote the phenotype of the parental lines and two additional genotypes (mean values ± standard error).

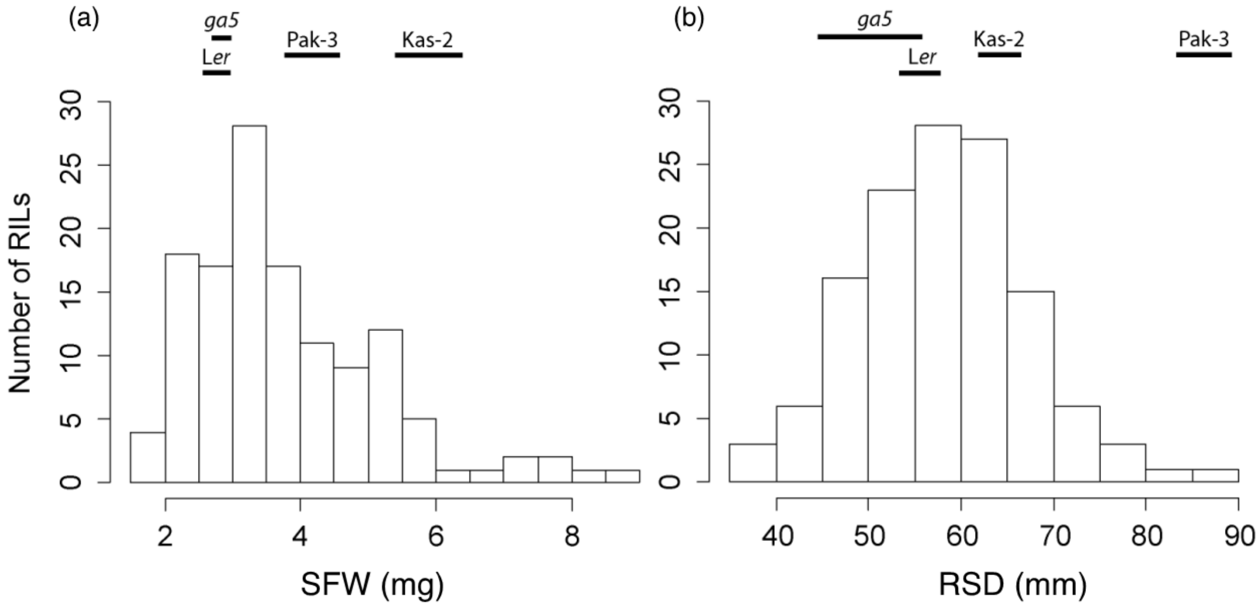

Fig. S5. Mean values of shoot fresh weight (SFW) and root system depth (RSD) in the L*er* × Kas-2 mapping population sorted by the parental alleles using the markers ERECTA, GA5 and SNP304. The phenotypes were evaluated in 125 recombinant inbred lines; six seedlings were sown per genotype. Means ± standard errors are shown.

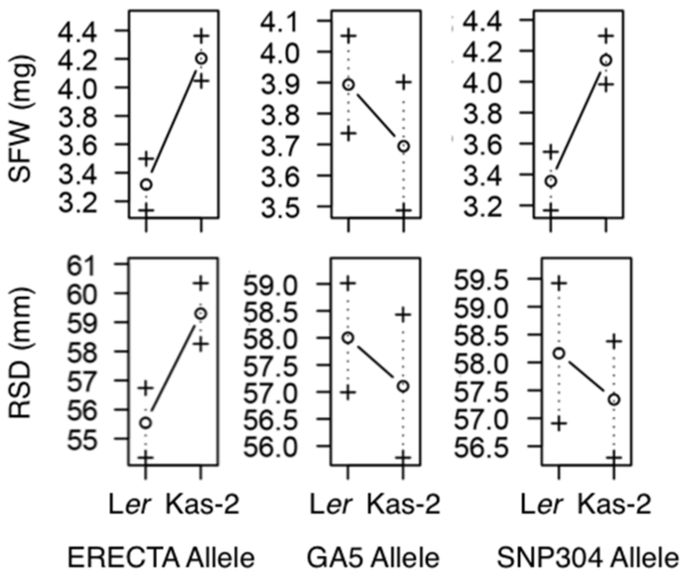

Fig. S6. QTL maps for shoot fresh weight (SFW) and root system depth (RSD) in the L*er* × Kas-2 mapping population grown *in vitro*. The phenotypes were evaluated in 125 recombinant inbred lines; six seedlings were sown per genotype. The medium contained: 0.8% Agar, 1/2 MS, pH 5.8. The experiment was evaluated 21 days after germination. The horizontal dotted line shows the significance threshold by running 1000 permutations. Position of the GA5 marker is indicated with an arrow.

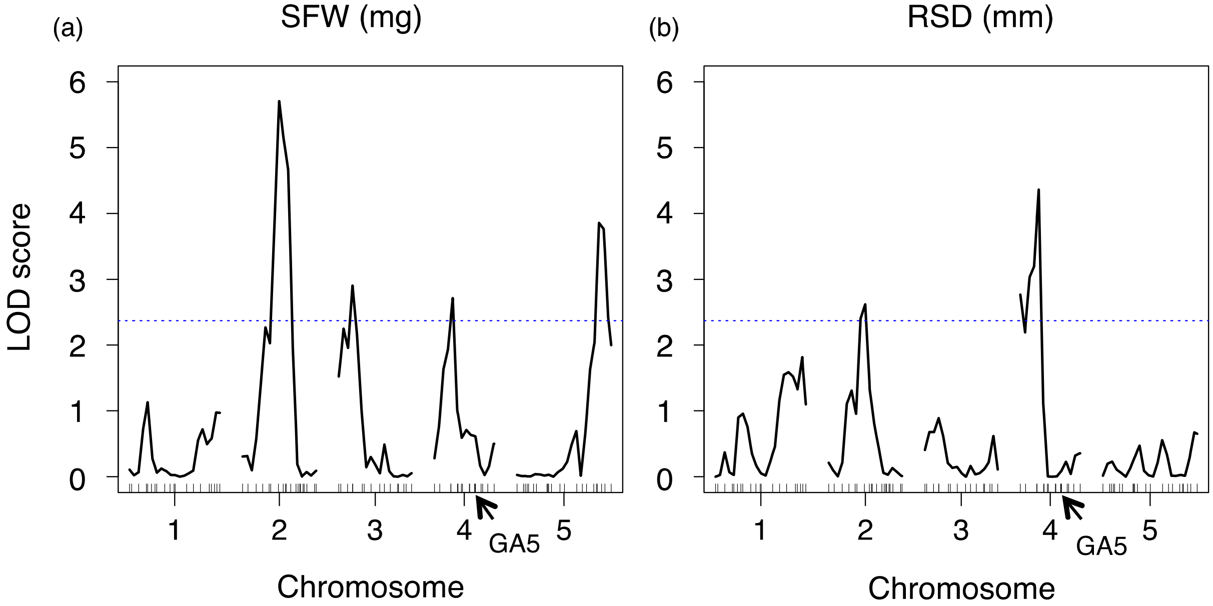

Fig. S7. Long Pak-3 root system depth occurs independently from the *ga5* inactive allele. Correlations between plant height (scored two weeks after flowering in greenhouse conditions) and root system depth (RSD, scored 28 days after sowing *in vitro*, medium 0.8% Agar, 1/2 MS, pH 5.8) in different F2 populations (+, L*er* × Pak-3 $n = 270$, *ga5* × Pak-3 $n = 250$), L*er* (filled square, $n = 22$), *ga5* (filled triangle, $n = 17$), and Pak-3 (filled circle, $n = 20$).

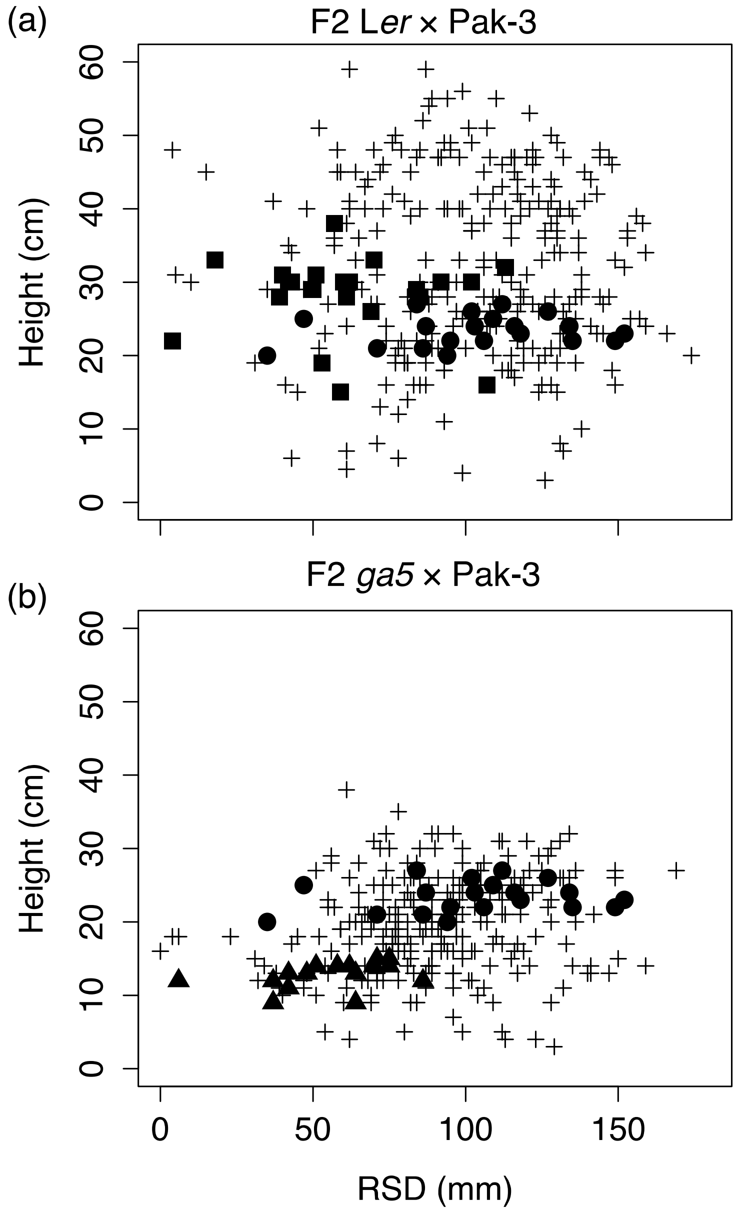

Fig. S8. Osmotic stress response (growth percentage under sorbitol relative to control) among *A. thaliana* accessions. a) Growth percentages for shoot fresh weight (SFW) and b) root system depth (RSD) representing variation among genotypes. Seedlings were grown *in vitro* (1% Agar with 1/3 Hoagland, evaluated 18 days after germination). Growth percentages were obtained using mean values (sorbitol/control) ± propagation of uncertainties (n = 3 – 11 for sorbitol, n = 6 – 12 for control). Vertical dotted lines separate semi-near isogenic comparisons. The symbol ⊙ indicates semi-dwarf accessions.

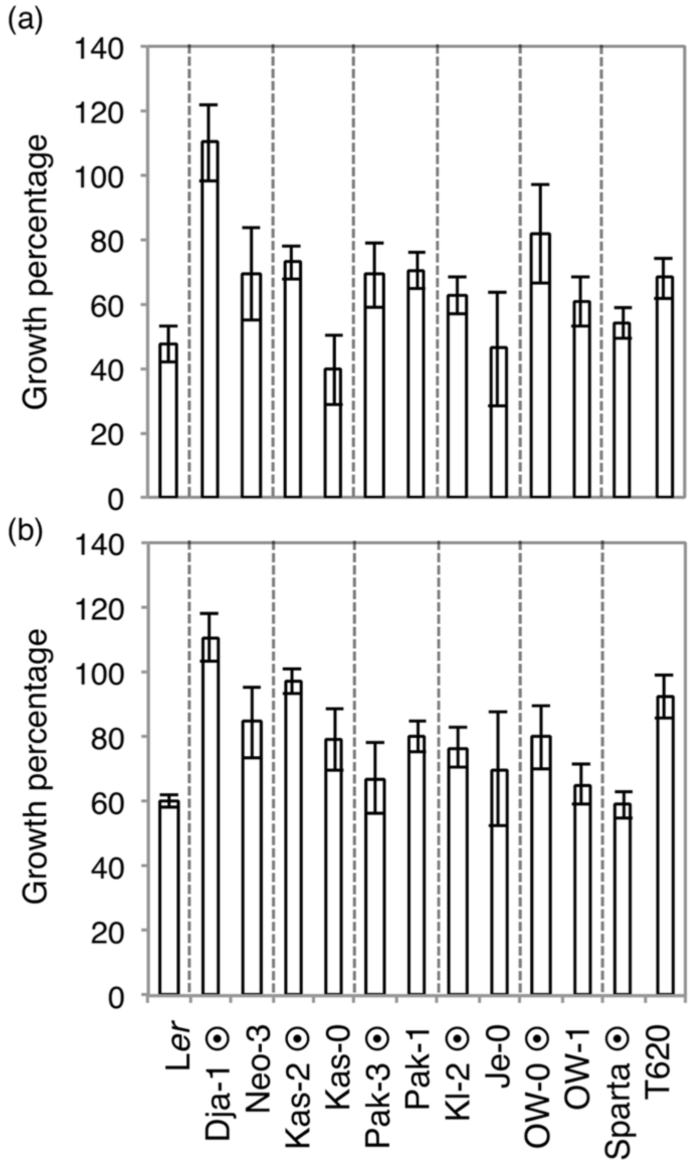

Supplement: Supplementary Data [file supp_mcv099_aob-15208-s01.pdf]
